# Brewing and biochemical characterization of *Camellia japonica* petal wine with comprehensive discussion on metabolomics

**DOI:** 10.1186/s43014-022-00109-w

**Published:** 2022-11-18

**Authors:** Soumya Majumder, Arindam Ghosh, Sourav Chakraborty, Malay Bhattacharya

**Affiliations:** 1 Molecular Biology and Tissue Culture Laboratory, Department of Tea Science, University of North Bengal, Siliguri, Darjeeling, West Bengal 734013 India; 2 Postgraduate Department of Botany, Darjeeling Government College, Darjeeling, West Bengal 734101 India

**Keywords:** Camellia, Quinic acid, GC-MS, Fermentation, Wine

## Abstract

A novel wine has been developed from *Camellia japonica*’s petal by fermenting the decoction with *Saccharomyces cerevisiae* or brewer’s yeast. pH, brix, specific gravity and alcohol percentage were tested to study the physicochemical properties of the wine. Qualitative tests indicated presence of phenols such as flavonoids, coumarins; protein; glycosides; glycerin; terpenoids; steroids; and fatty acids in the wine. Total phenol content was found high in the decoction and in its fermented form as well. In vitro biological activities such as antioxidant activity, antidiabetic activity and lipid peroxidation inhibition power were assessed in samples. Furthermore, GC-MS analysis helped to detect volatiles present in the unfermented decoction and understand the effect of fermentation on its changing metabolome while column chromatography assisted the separation of solvent-based fractions. Notable outcomes from this study were detection of bioactive compound quinic acid in the decoction and a proposed pathway of its metabolic breakdown after fermentation. Results of this research revealed biochemical and physicochemical acceptability of this wine prepared from an underutilized flower.

**Graphical Abstract:**

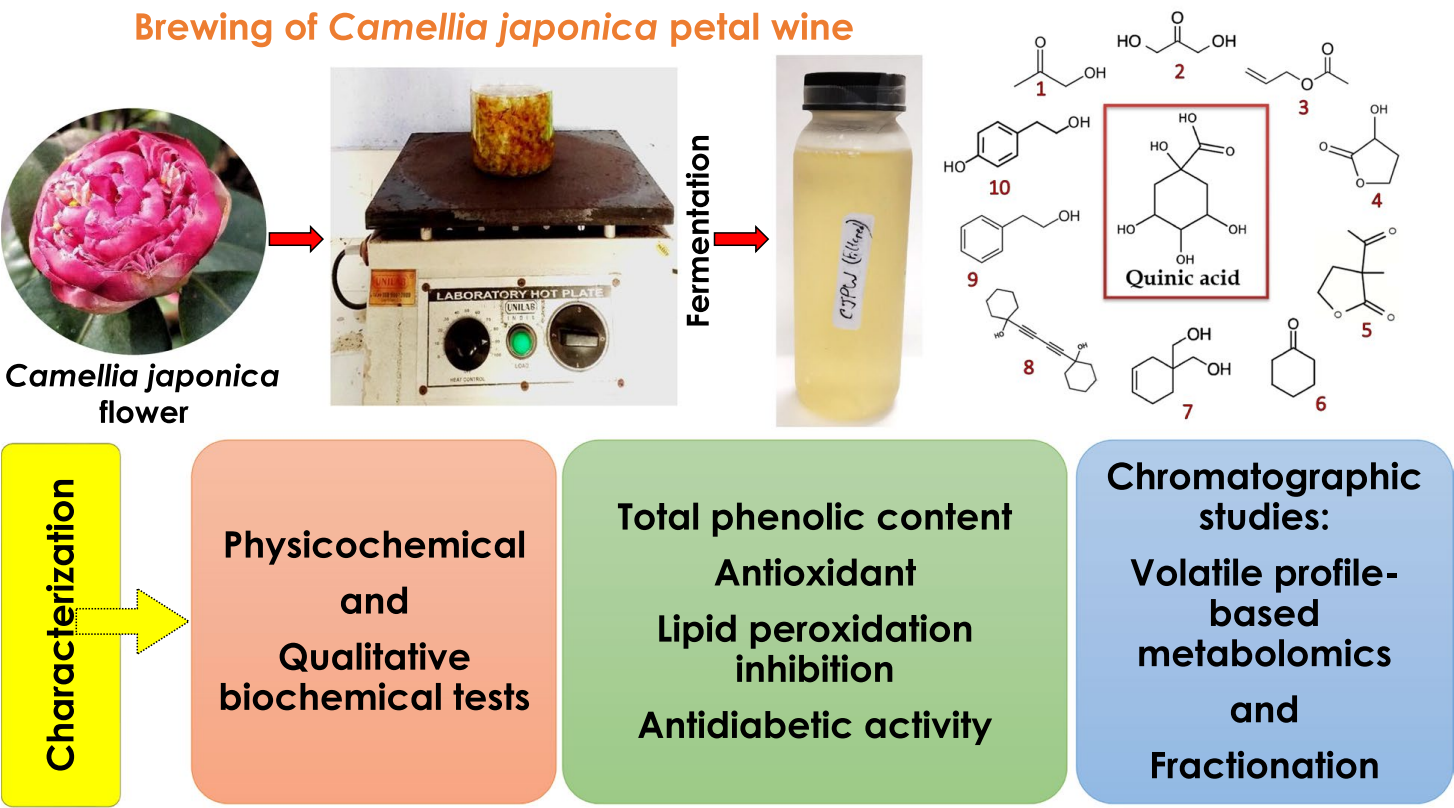

## Introduction

Wine brewing is considered as an important part of fermentation science and food technology where cereals, fruits, leaves, edible flowers etc. from various plants are used as raw materials. Wines contain alcohols; fatty acids; terpenoids; polyphenols such as flavonoids, anthocyanins, flavonols, catechins; and other secondary metabolites (Majumder, Saha, et al. 2021) which exhibit a wide range of biological activities including antioxidant property. Catechin and their oligomers, proanthocyanidins, quercetin, resveratrol and other polyphenols of wine are health protective agents exhibiting cardioprotective, anti-carcinogenic, anti-diabetic, anti-atherogenic, anti-inflammatory, antiviral and antibacterial activities (Banc et al. 2014). Typically, *Saccharomyces cerevisiae* or brewer’s yeast is used as fermentation starter by the western world, while the Asian scenario is different. Asians rely on their traditional expertise where fermentation of foods and beverages are carried out using various microbial cultures such as old cultures of yeast, filamentous molds, bacteria, symbiotic cultures of bacteria and yeasts etc. In fermentation process, utilization of substrate depends on availability and productivity of cultivated grain crops and fruits of a region. There are a number of such region specific fermented beverages, for example; red wine and white wine from grape producing European countries like Italy, France, Spain and Germany; probiotic fermented tea kombucha from largest tea producer China; wheat based fermented alcohol vodka from Russia; rice based beverages like haria, apong, chhyang, zutho, lugdi etc. from India; traditional alcoholic beverages like sake and shochu of Japan which are prepared fermenting substrates like sticky-rice, millet and satsumaimo (Japanese sweet potato); rice based traditional fermented beverage-Brem from Indonesia; South African alcoholic beverage amarula prepared from African marula (*Sclerocarya birrea*) fruits; pomegranate wine from Israel; Filipino Tuba (coconut wine); lychee and cherry wines from China etc.

Flowers such as, rose, China-rose, dandelion, marigold, lavender, gorse, mahua etc. are also used as potential brewing substrates traditionally or industrially. In the rhododendron growing regions of the Himalayas, guras (wine from rhododendron’s flower petal) is a popular drink among the local inhabitants and tourists. Flower wines are often praised by consumers as well as oenologists for unique flavors and medicinal properties (Majumder, Saha, et al. 2021). Recently, our research group have produced a wine from petals of tea or *Camellia sinensis* and successfully validated the biochemical and physicochemical acceptability of that fermented product through in vitro experiments and volatile screening (https://fppn.biomedcentral.com/articles/10.1186/s43014-021-00075-9). Moreover, that research work inspired us to brew a wine from another underutilized edible flower i.e., *Camellia japonica* which is a plant of the same family as of tea i.e., Theaceae or “tea family”. In the wild, this plant grows as an understory plant on hillsides at altitudes of around 300–1100 m (http://www.efloras.org/florataxon.aspx?flora_id=2&taxon_id=200014034). Its botanical origin is in Eastern Asia (Japan) and also found in the Himalayas, Southern China, and other South-Eastern Asian countries (Pereira et al. 2022). Presently, it is planted in households and gardens as an ornamental plant throughout tropical and subtropical regions. Leaf, seed oil, and bark of *Camellia japonica* are reported to exhibit anti-inflammatory (Lim 2014) and other medicinal properties because of several bioactive components like vitamin E, n-eicosane, neophytadiene; squalene and its derivatives; n-octacosane; 6,9-pentadecadien-1-ol, α -linolenic acid and n-hexadecanoic acid (Majumder et al. 2020; Yoon et al. 2017). Being anti-inflammatory, antiviral (Yang et al. 2015; Yoon et al. 2017) and rich in phenolic contents (Lee et al. 2011; Li et al. 2014) flowers of this plant are also consumed in eastern parts of Asia (https://pfaf.org/user/Plant.aspx?LatinName=Camellia+japonica). Regarding metabolomics, different organs of this plant have been studied by various researchers for a long period of time (Majumder et al. 2020). Flower buds of this plant has already been reported bioactive for showing antioxidant properties due to presence of high phenolics (Lee et al. 2011; Li et al. 2014). Recently, Kim et al. (2019) has demonstrated antiradical effects of *Camellia japonica* flower extract and they also proposed that it can be used as a protective material against urban air pollutant-induced skin damages. However, till date, application of this plant in life sciences (biotechnology, food science, pharmacology etc.) is limited upto exploring anti-inflammatory properties of it where the flower is underutilized due to lack of appropriate research.

This research was aimed to introduce *Camellia japonica* flower in fermentation technology to utilize it for brewing wine. Objectives of this research were to produce a wine from *Camellia japonica* petals, to analyze the antioxidant potential of the product through various in vitro experiments; to explore the volatile profile by GC-MS analysis; and to study the fermentation metabolomics.

## Materials and methods

### Collection of *Camellia japonica* flowers and brewing ingredients

**Fig. 1:**
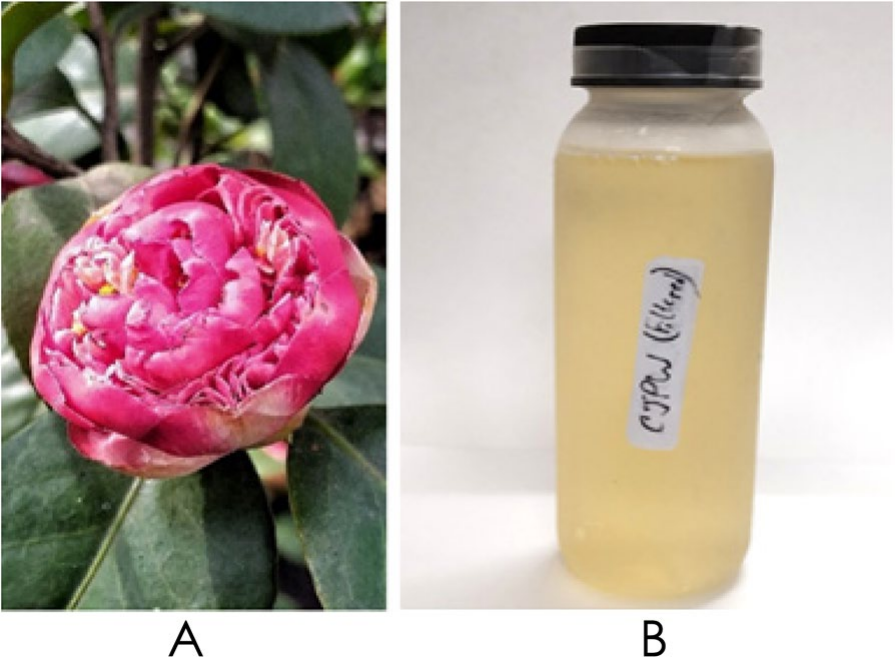
**A** A freshly bloomed *Camellia japonica* L. flower, **B** *Camellia japonica* petal wine or CJPW (filtered and bottled after 3 months of aging)

A healthy and matured *Camellia japonica* plant was selected from organically grown in-pot propagated cuttings which were previously obtained from a mother bush. A flowering twig of the plant was deposited in Kalimpong College Herbarium (Voucher no. KPGC/MB/85A) and the specimen was properly identified as *Camellia japonica* L. by botanists of Kalimpong College (West Bengal, India). Freshly bloomed flowers (Fig. 1A) were harvested from the tree whose petals were immediately removed, washed with tap water and used to prepare the decoction. Other brewing ingredients such as, starter i.e., dry brewer’s yeast (Goodrich, India); sucrose (Sulphur free sugar, Uttam sugar, India); and synthetic white vinegar (William chemical co., India) were bought from local market.

### Brewing wine from decoction of petals

Following the petal wine brewing protocol of Majumder, Saha, et al. (2021) *Camellia japonica* petal wine (Fig. 1) was prepared with slight modifications. *Camellia japonica* petals (30 g) were added to one liter of sterile distilled water and boiled for 20 min to prepare *Camellia japonica* petal decoction or CJPD. Decoction was filtered through sterile muslin cloth into an autoclavable jar. Sucrose (30 g) was added as an additional carbon source or nutrition for fermenting yeasts. Then, the broth or decoction was autoclaved and left for cooling at room temperature (25 ± 1 °C). After cooling, pH of the broth was measured, and sterile synthetic white vinegar was added to lower the pH around 5 to create an acidic environment and controlled condition for fermentation. Two grams of dried brewer’s yeast were inoculated as starter in the jar. Mouth of the jar was then covered with sterile polythene where pores were made with a sterile steel needle to facilitate the release of fermentation derived CO_2_. Following this procedure, triplicates were prepared and left for 15 days in a dark room at 25 ± 1 °C for incubation to produce *Camellia japonica* petal wine (CJPW). Similarly, a control batch of wine (CW) was prepared without using petal where the petal decoction was replaced with double distilled water. *Camellia japonica* petal wine or CJPW and control wine or CW (after 15 days of incubation) and freshly prepared decoction or CJPD were taken as samples for further experiments. Moreover, CJPW (Fig. 1B) was left for 3 months to study the changes in its properties during aging.

### Physicochemical properties

Physicochemical parameters such as, optical density (OD), transmission percentage (%T), pH (for acidity), brix (%Bx) and alcohol by volume (%ABV) etc. were measured in the samples following the protocol of Majumder et al. (2022a) to study the fermentation driven alteration in those characteristics and analyze them in a comparative way. OD and %T at 420 nm were measured using a UV-vis spectrophotometer (Cary-60, Agilent). pH by using a calibrated pH meter (LMPH10, Labman Co.) to investigate the changes in acidity of the broth due to fermentation. A pre-calibrated brix meter (0–90% RHB-55 Brix ATC refractometer, Labart) was used to measure the %Bx. Alcohol percentage or ABV in the fermented samples was calculated from Brix results using an ABV calculator (https://www.petedrinks.com/abv-calculator-refractometer-hydrometer/). All the experiments were carried out on both CJPW and CW to study the changes in physicochemical properties during fermentation. Each result has been expressed as mean ± SD (*n* = 3).

### Qualitative biochemical tests

Qualitative tests were conducted on all samples i.e., CJPD, CJPW and CW to determine the presence of different groups of molecules such as phenols, flavonoids, coumarin, protein, starch, glycosides, glycerin, terpenoids, steroids (Das et al. 2020; Labar et al. 2019; Majumder, Acharyya, et al. 2021) and alkaloid (https://www.biologydiscussion.com/plants/detection-of-alkaloids-plants/57192). Results were noted in a comparative approach to prepare a heatmap. Qualitative test for free fatty acid was done following phenolphthalein test developed by Cox and Pearson (1962). Results of this experiment helped to study the alteration of bio-chemical profile of the decoction due to fermentation.

### Total phenolic content (TPC)

Total phenolic content (TPC) in samples (CJPD, CJPW and CW) were determined by the Folin–Ciocalteu method (Blainski et al. 2013). Two hundred microliters of each sample was taken and made up to 3 ml with distilled water, mixed thoroughly with 500 μl of Folin–Ciocalteu reagent (SRL, India) for 3 minutes, followed by the addition of 2 ml of 20% (w/v) sodium carbonate. The mixture was allowed to stand for a further 60 min in the dark, and absorbance was measured at 650 nm in a UV-vis spectrophotometer (Cary-60, Agilent). TPC was measured against gallic acid standard curve (*R*^2^ = 0.9975; y = 0.0043x - 0.1672) and results were expressed as gallic acid equivalent (mg GAE/100 ml).

### In vitro antioxidant activity

#### DPPH assay

Protocols of Chakraborty, Majumder, Ghosh, Saha, and Bhattacharya (2021) and Majumder, Saha, et al. (2021) were followed in this assay. Antioxidant activity (scavenging percentage) was determined from the decreasing absorbance of 100 mM DPPH (HiMedia, India) solution (2800 μl) due to exposure of sample (200 μl) at 517 nm. Result of this assay was expressed as mean ± SD (*n* = 3). Beside this conventional experiment i.e., quantification of antioxidant value, DPPH assay was also applied to study the “DPPH inhibition kinetics” (Chakraborty, Majumder, Ghosh, Saha, & Bhattacharya 2021; Majumder et al. 2022b) to evaluate the inhibition power graphically. DPPH inhibition kinetics is an observational analysis suitable for comparative studies. A kinetic analysis for 30 minutes was programmed in UV-vis spectrophotometer for this assay.

#### Iodometric assay

Antioxidant activity of the samples (CJPD, CJPW and CW) were also determined using this titrimetric assay. According to experimental procedure, 1 ml of 1% starch indicator was added in each sample solution (20 ml of sample dissolved in 150 ml of distilled water) which was titrated with 0.005 mol/L iodine solution (https://uwaterloo.ca/chem13-news-magazine/september-2015/activities/sharing-chemistry-community-c-difference). The redox titration endpoint was determined by the first iodine excess that is complexed with starch, giving a deep blue-violet color. As long as there is any antioxidant or reducing agent, the iodine is reduced to iodide ions which apparently arrests the formation of starch-iodine complex (https://www.canterbury.ac.nz/media/documents/science-outreach/vitaminc_iodine.pdf). Ascorbic acid was used as a calibration standard (*R*^2^ = 0.9999; y = 3.9022x - 0.206) to quantify the values of reducing power shown by samples. Results have been expressed as μg AAE/ ml (microgram ascorbic acid equivalent per milliliter).

### In vitro lipid peroxidation inhibition assay

The in vitro lipid peroxidation inhibition assay was done following Rahman et al. (2015). Value of estimated lipid peroxidation inhibition activity was calculated referring a vitamin E or (tocopheryl acetate) standard curve (*R*^2^ = 0.9973; y = 0.0077x + 0.0321). Results have been expressed as mg TAE/ml (milligram tocopheryl acetate equivalent per milliliter). In this research, goat liver homogenate was used to evaluate lipid peroxidation inhibition activity, so, hepatoprotective efficacy was also determined indirectly through this simple in vitro assay.

### In vitro antidiabetic activity

Glucose uptake capacity by yeast cells was determined for this assay (Shettar et al. 2017). Metronidazole (Abbott, U.S.) was used as reference to prepare the standard curve (*R*^2^ = 0.9862; y = 0.0119x - 0.0003). Results were expressed as mg MetE/ml (milligram Metronidazole Equivalent per milliliter).

### Gas chromatography-mass spectrometry analysis

All the three samples (CJPD, CJPW and CW) were subjected to GC-MS analysis following the pre-standardized research protocol for tea petal wine developed by Majumder, Saha, et al. 2021. One milliliter of each sample was air dried and dissolved in 1 ml methanol to prepare methanolic extract prior to GC-MS analysis. GCMS-QP2010 Plus (Shimadzu Co., Japan) having a DB-5 fused-silica capillary column (30 m × 0.25 mm × 0.25 μm) was used. One microliter of each centrifuged sample was split injected with a ratio of 20:1. Injection temperature was 260 °C and interface temperature was 270 °C. Ion Source temperature was adjusted to 230 °C. Helium gas (99.9%) was used as carrier. Total flow rate and column flow rate were 16.3 ml/min and 1.21 ml/min respectively. Mass spectra were recorded at 5 scan/sec with a scanning rate of 40–650 m/z. The compounds were identified after comparing the spectral configurations obtained with that of the available mass spectral database. TIC or Total Ion Chromatogram was formed based on the intensity of fragments produced by the ionization. Peak area percentage (%) was considered for quantification of the amount of each compound detected.

### Fractionation of CJPW and antioxidant activity

CJPW (5 ml) was dried and put in a silica gel (Merck, 200–400 mesh size) loaded column where a series of nonpolar to polar solvents (hexane, toluene, chloroform, diethyl ether, ethyl acetate, acetone, ethanol, methanol and water) were passed to fractionize the sample on the basis of molecular affinity towards solvents of different polarities (Bhattacharya et al. 2009). Each fraction was reduced/ volumed up to 5 ml with methanol. DPPH free radical scavenging potential of CJPW fractions was evaluated. This cumulative experiment of chromatography and antioxidant activity validated the separation of potential components responsible for this activity.

### Aging dependent alterations in physicochemical properties and antioxidant activity of *Camellia japonica* petal wine (CJPW)

After completing 15 days of fermentation and experiments, CJPW was left in fermentation jars upto 3 months for aging. Following the same protocols described above, physicochemical properties (pH, %Bx, and %ABV) and antioxidant activity (DPPH assay) of the sample CJPW were determined on regular intervals upto 3 months of aging to investigate the alteration of qualities of the prolongedly fermented wine. Specific gravity was determined by calculating the ratio of the specific weight of sample to the specific weight of water at 4 °C (https://scetcivil.weebly.com/uploads/5/3/9/5/5395830/fluids_chap01.pdf).

### Data analysis

Data obtained from the various experiments during this research were analyzed statistically using Microsoft Excel. The data were expressed as means of replicate ± Standard Deviation (SD). The test for statistical difference was performed using one-way ANOVA (analysis of variance). Differences were considered significant at *P* < 0.05.

GC-MS detected compounds were identified from their mass-spectra by comparing the mass-spectra found in library databases (NIST08s.LIB and WILEY8.LIB). List of peak reports were then prepared with the names of the compounds detected. Chemical classification of each compound was based on ChEBI (Chemical Entities of Biological Interest) ontology (https://www.ebi.ac.uk/chebi/) and PubChem database (https://pubchem.ncbi.nlm.nih.gov/). The data obtained from GC-MS analysis were further studied from available literature to analyze the fermentation metabolomics (Majumder, Ghosh, et al. 2021). The compounds detected by GC-MS were matched to The Good Scents Company database or TGSC (http://www.thegoodscentscompany.com/) to study their impact on flavour and taste imparting properties (Chakraborty, Majumder, Ghosh, & Bhattacharya 2021).

## Results

### Physicochemical properties

After 15 days of fermentation, OD of samples was seen to be decreased (58 ± 2 to 95 ± 1; and 65 ± 1 to 87 ± 4) and %T was increased (0.237 ± 0.015 to 0.022 ± 0.004; and 0.187 ± 0.007 to 0.060 ± 0.02) in CJPW and CW respectively. A pH of 5.32 ± 0.28 was recorded for CJPD. On day 0, the pH of CJPW and CW was lowered using vinegar and maintained at 5 to level the acidity of broths before initiation of fermentation process. Furthermore, this pH level of the broths was again decreased after fermentation (3.89 ± 0.19 and 4.19 ± 0.2 for CJPW and CW respectively). Alcohol percentage for CJPW was calculated to be 8.31 ± 0.47% as its Brix was dropped from 19.5 ± 0.2 to 5.9 ± 0.3. In CW, 5.19 ± 0.12% of ABV was measured as Brix was changed to 2.5 ± 0.4 from 12.1 ± 01 after 15 days.

### Qualitative biochemical tests

**Fig. 2:**
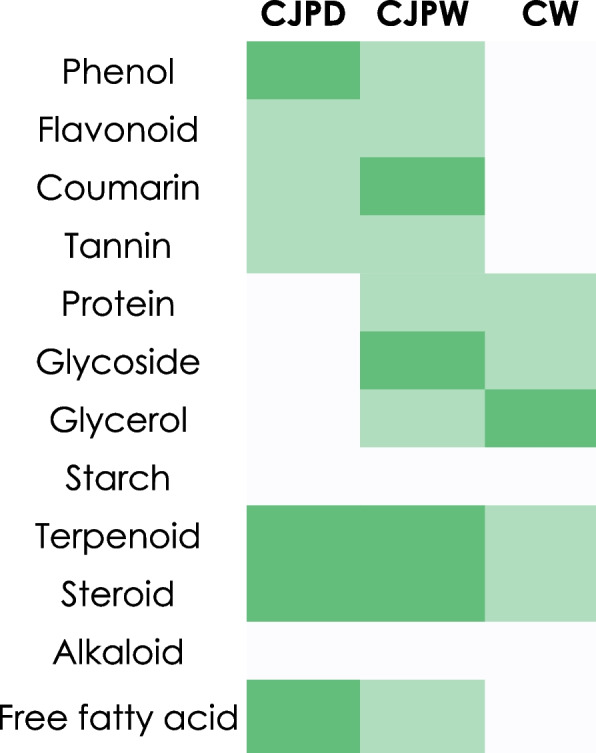
Heatmap (Dark green – white = high – low) showing comparative results of qualitative biochemical tests

Qualitative biochemical tests revealed the presence of phenol, flavonoid, coumarin and tannin in both CJPD and CJPW while CW was found to be devoid of most of the qualities except one fermented product i.e., glycerin which was detected higher in CW compared to CJPW. Terpenoid and steroid were detected in all three samples through respective indication tests. A heatmap (Fig. 2) has been added to express the results.

### Total phenolic content (TPC)

**Fig. 3:**
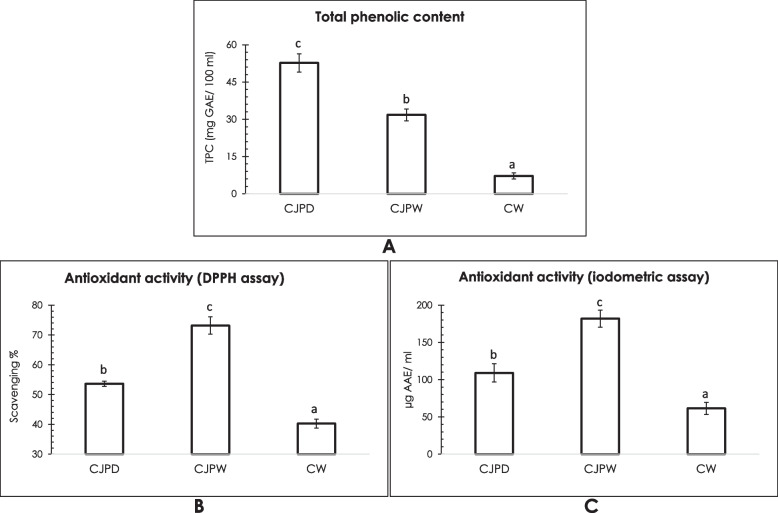
**A** Results of total phenolic content or TPC; **B** Results of in vitro antioxidant activity (DPPH assay); and **C** Results of in vitro antioxidant activity (iodometric assay). Values are means ± standard deviation, means with different superscripts in each figure are significantly different (*P* < 0.05). GAE: gallic acid equivalent; AAE: ascorbic acid equivalent

The TPC of the decoction or CJPD (100 ml) was quantified to be 52.698 ± 3.66 mg GAE and the value was tested to be decreased (31.775 ± 2.36 mg GAE/100 ml) in the wine sample (CJPW). Demonstrating phenols as usual fermentation metabolites of wine yeasts, CW also exhibited a considerable amount of phenolics (7.18 ± 1.22 mg GAE/100 ml) but it was nowhere near the petal samples. A graphical representation of this result has been given in Fig. 3A.

### In vitro antioxidant activity

CJPW showed highest DPPH scavenging activity (73.16 ± 2.9%) followed by the unfermented decoction or CJPD (53.63 ± 0.87%) while the control one (CW) showed the least activity with 40.25 ± 1.5%. Figure 3B is a graphical representative of results of this assay.

Iodine reducing power of CJPW was calculated to be 181.79 ± 11.41 μg AAE/ ml which was also higher than others (Fig. 3C) i.e., unfermented CJPD (109.12 ± 12.2 μg AAE/ ml) and CW (61.35 ± 8.05 μg AAE/ ml).

**Fig. 4:**
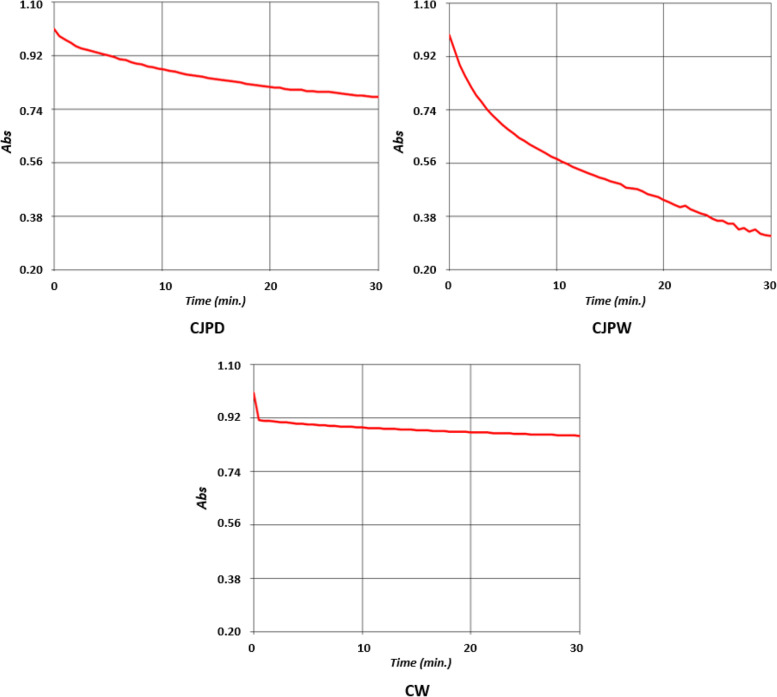
Results of DPPH inhibition kinetics assay showing a prominent and faster rate of DPPH inhibition by fermented petal wine (CJPW)

Figure 4 represents the results of DPPH inhibition kinetics assay. Graph of fermented petal wine (CJPW) exhibited a sharp and prominent decline demonstrating a significant faster rate of inhibition compared to the slightly curved graph of unfermented CJPD. Slightest decline was noticed in CW’s graph.

### In vitro lipid peroxidation inhibition assay

**Fig. 5:**
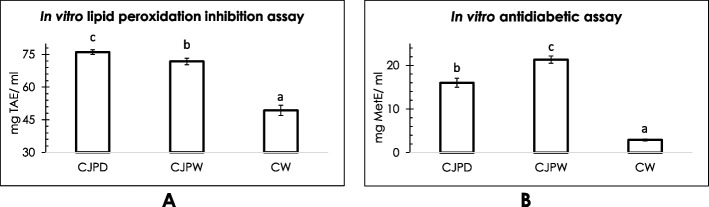
Results of in vitro lipid peroxidation inhibition assay (**A**) and in vitro antidiabetic activity (**B**). Values are means ± standard deviation, means with different superscripts in each figure are significantly different (*P* < 0.05). TAE:  tocopheryl acetate equivalent; MetE: metronidazole equivalent

Lipid peroxidation is a series of oxidative degradation reaction occurred with lipid molecules. It is the process in which free radicals collect electrons from the lipids in cell membranes, resulting in oxidative damage. Inhibition of this lipid peroxidation level by 1 ml of CJPD was estimated to be equivalent to 76.06 ± 1.1 mg tocopheryl acetate. For fermented wine sample, the value was slightly lower with 71.8 ± 1.5 mg TAE/ml (Fig. 5A).

### In vitro antidiabetic activity

Glucose uptake capacity by yeast cells was assessed spectrophotometrically to evaluate antidiabetic activity. One milliliter of CJPD showed antidiabetic activity that was equivalent to 16.02 ± 1.04 mg of metronidazole while the wine or CJPW resulted better activity (21.33 ± 0.81 mg MetE/ ml) (Fig. 5B).

### Gas chromatography-mass spectrometry-based metabolomics

**Fig. 6:**
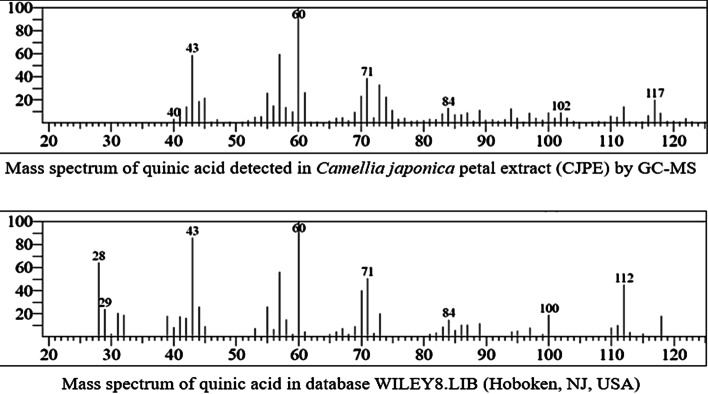
Mass-spectrum of quinic acid (most abundant component with 45.9% peak area) in sample CJPD

Metabolites of CJPD, CJPW and CW detected by GC-MS analysis have been listed in Table 1. Total 36 compounds belong to different chemotaxonomy and various biosynthesis pathways were present in CJPD including mostly of known bioactive compounds, where quinic acid was major and most abundant with 45.9% peak area (resulting mass spectrum is given in Fig. 6). GC-MS analysis of fermented samples revealed glycerin as a major compound. The fermentation-lead biotransformation of quinic acid has been elucidated in the “Discussion” section.

**Table 1:** Comparative table for metabolites (volatile profile) of CJPD, CJPW and CW detected by GC-MS analysis

Chemical family	Name of compounds	Peak Area %		
		CJPD	CJPW	CW
Monoterpenoid	1,2-diisopropenylcyclobutane	0.1	–	–
	Eucalyptol	0.26	–	0.08
	alpha-Terpinyl acetate	0.19	–	–
	L-Camphor	–	–	0.18
Sesquiterpenoid	alpha-Gurjunene	0.74	–	–
	beta-Caryophyllene	0.36	–	–
	gamma-Muurolene	0.39	–	–
	delta-Cadinene	0.07	–	–
	2,4a,8,8-tetramethyl-decahydro-cyclopropa[D]naphthalene	0.36	–	–
Diterpenoid (esterified)	Methyl isodextropimarate	0.2	–	–
Triterpenoid	Squalene	0.91	–	–
	Chondrillasterol	1.46	–	–
Tocopherol	Vitamin E	0.57	–	–
Fatty acid derivatives	Methyl 3-hydroxyhexanoate	31.4	–	–
	Dimethyl 3,8-dioxodecanedioate	0.19	–	–
	Methyl palmitate	1	–	–
	2-Chloroethyl linoleate	0.35	–	–
	Dimethyl 4-methylheptanedioate	0.1	–	–
	Dodecyl cis-9,10-epoxyoctadecanoate	0.1	–	–
	Diethyl succinate	–	–	0.11
Fatty alcohol	Arachidyl alcohol	0.66	–	–
Fatty aldehyde	Valeraldehyde	–	3.66	–
Long chain hydrocarbons or wax	Hentriacontane	0.21	–	–
	Oxirane, hexadecyl-	0.24	–	–
	Tetratetracontane	0.32	–	–
	Pentacosane	1.23	–	–
	Docosane	0.32	–	–
	Erythro-9,10-dibromopentacosane	0.61	–	–
	Tetracontane	1.87	–	–
	Hexatriacontane	0.7	–	–
	(Z,Z)-6,9-cis-3,4-epoxy nonadecadiene	0.49	–	–
	Docosanal	0.15	–	–
Primary alcohol	1-Hydroxyacetone	–	2.96	–
	Dihydroxyacetone	–	11.55	–
	Phenylethyl alcohol	–	3.03	1.73
Secondary sugar alcohol	2,3-Butanediol	–	0.98	7.52
	3-Pentanol, 2,3,4-trimethyl-	–	1.19	–
	2,5-Methylene-d,l-rhamnitol	–	1.34	–
	2,5-Monomethylene-l-rhamnitol	–	12.43	–
Sugar aldehyde	Methylglyoxal	–	13.25	–
	Glyceraldehyde	–	2.88	–
Glycol	Phenylethylene glycol	–	–	0.3
Triol	1,2,3-Butanetriol	–	1.6	–
	Glycerin	–	16.88	86.43
Glycerin derivative	cis-5-Hydroxy-2-methyl-1,3-dioxane	–	0.27	1.27
	4-Hydroxymethyl-2-methyl-1,3-dioxolane	–	–	0.5
Acetate (esterified)	Acetic acid, 2-propenyl ester	–	2.46	–
Pyrone (flavonoid fraction)	2,3-Dihydro-3,5-dihydroxy-6-methyl-4H-pyran-4-one	4.37	0.83	–
Phenolics	Quinic acid	45.9	–	–
	Tyrosol	–	1.11	–
Furanone derivatives	2-Hydroxy-gamma-butyrolactone	–	13.33	–
	2-Acetyl-2-hydroxy-gamma-butyrolactone	–	0.27	–
	5-Acetyldihydrofuran-2(3H)-one	–	–	0.66
	4-(1-hydroxyethyl)-gamma-butyrolactone	–	–	0.51
Ketoxime	2,3-Butanedione monoxime	–	1.85	–
Cyclohexanone derivatives	Cyclohexanone	–	0.58	–
	3-Cyclohexene-1,1-dimethanol	–	1.02	–
	1,1′-Butadiynylenedicyclohexanol	–	2.56	–
Lactone	3-Deoxy-d-mannoic lactone	–	2.33	–
	Ethylene brassylate	1.56	–	–
alpha-Amino acid (esterified)	Methyl L-pyroglutamate	–	0.51	–
Dipeptide	Dimethylamine, n-(neopentyloxy)-	0.08	–	–
Phthalates	Phthalic acid	1.26	–	–
	Diethyl phthalate	0.61	1.14	0.71
	Tricyclo[4.3.0.0(7,9)]nonane, 2,2,5,5,8,8-hexamethyl-, (1.alpha.,6.beta.,7.alpha.,9.alpha)	0.6	–	–

### In vitro antioxidant activity of CJPW fractions

**Fig. 7:**
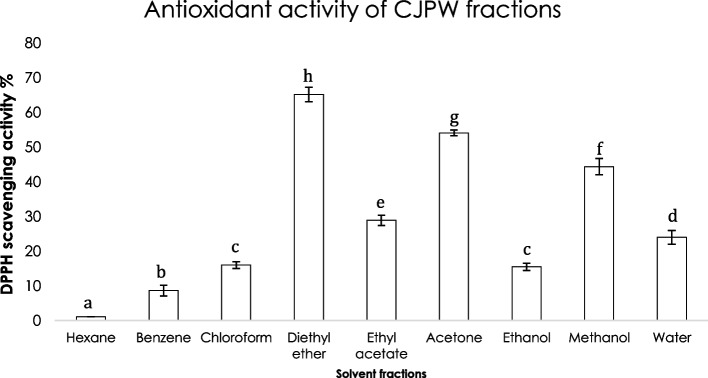
Antioxidant activity (DPPH scavenging activity %) of different solvent fractions of CJPW (*Camellia japonica* petal wine). Error bars represent standard deviations. Means with different lowercase superscript alphabets differ significantly (*P* < 0.05)

Result of this experiment revealed that activity shown by crude wine sample was higher than its fractions. However, fraction obtained in diethyl ether solvent (65.16 ± 2.1%) followed by acetone (54.09 ± 0.82%) and methanol (44.36 ± 2.33%) fractions exhibited a moderate activity (Fig. 7).

### Aging dependent alterations in physicochemical properties and antioxidant activity of *Camellia japonica* petal wine (CJPW)

**Fig. 8:**
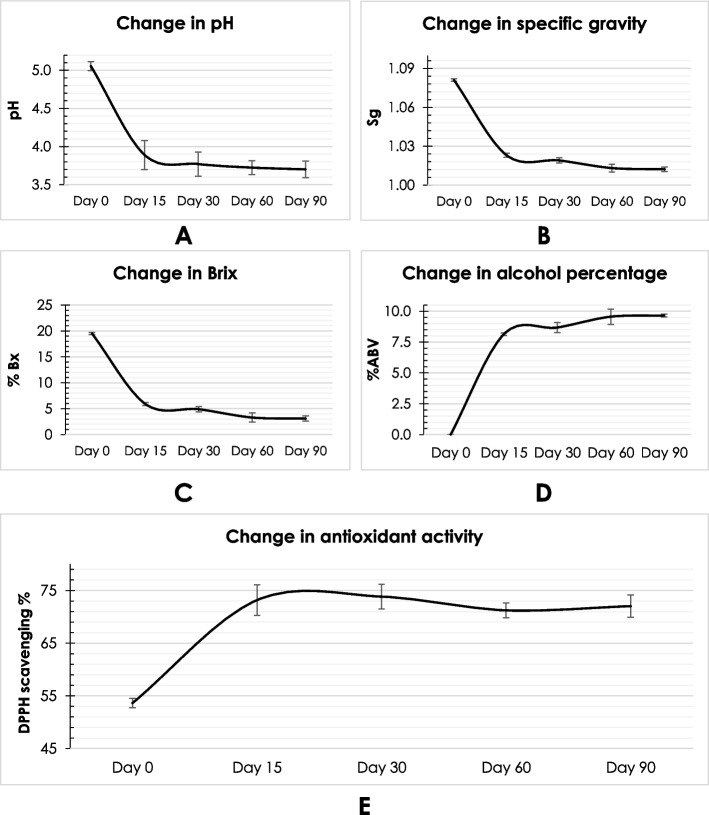
Alteration in properties of *Camellia japonica* petal wine (CJPW) during aging. **A** pH; **B** Specific gravity (Sg); **C** Brix (%Bx); **D** Alcohol by volume (%ABV); **E** Antioxidant activity (DPPH assay)

Aging of CJPW has introduced some changes in wine’s quality which was studied evaluating selected physicochemical parameters (pH, specific gravity, %Bx, and %ABV) and antioxidant (DPPH assay) property. Noticeable alterations were observed during aging mostly upto first 30 days of fermentation (Fig. 8). pH of CJPW was decreased from 5.06 ± 0.06 to 3.77 ± 0.16 after 1 month of aging signifying a fermentation lead acidification inside the broth. Any change in antioxidant activity (DPPH scavenging percentage) was hard to observe (Fig. 8E) but a slight upward graph was there in the one-month aged sample which was further stabilized. Successive decrease in specific gravity (from 1.081 ± 0.001 to 1.013 ± 0.003) and %Bx (from 19.5 ± 0.2 to 3.3 ± 0.9) were recorded during aging (upto 60 days) which indicated an increased rate of alcohol production (around 9.5% ABV). Alterations in specific gravity, Brix (%Bx) and alcohol by volume (%ABV) have been graphically represented in Fig. 8.

## Discussion

Physicochemical properties like OD, %T, pH, specific gravity, %Bx and %ABV are considered important characteristics to judge the rate of fermentation and physicochemical acceptability of any fermented beverage (Majumder, Saha, et al. 2021; Majumder et al. 2022b). Usually, a broth becomes gradually lighter in colour and transparent during fermentation except the surface area where yeast cells sometimes form a film (also called flor or kahm yeast) and the bottom portion where dead yeast bodies get sedimented as cell debris (Majumder, Sahaa, et al. 2021). Changes in OD and %T indicated these biological and chemical changes such as microbial growth, utilization of anabolites, microbial breakdown and production of secondary metabolites, changes in viscosity etc. during fermentation in studied samples.

Fermented foods or beverages are usually found to be acidic in nature. During fermentation of wines and probiotic beverages, yeasts and bacteria cause acidification by releasing organic acids as secondary metabolites which makes the broth acidic and accelerate the fermentation rate as well (Majumder et al. 2022b). pH of a typical wine ranges between 3 to 4 (Majumder, Saha, et al. 2021). Therefore, pH value of CJPW (3.89 ± 0.19) was quite acceptable even after 3 months of aging i.e., pH 3.7 ± 0.11. Rising acidic condition inside CJPW accelerated the fermentation rate which has been reflected by results of alcohol percentage and other physicochemical parameters (Fig. 8A).

Specific gravity and %Bx are factors to determine the desired level of viscidness and alcohol percentage and the point of acceptability of a wine. During fermentation, specific gravity and %Bx were found to be decreased in CJPW indicating an increased alcohol production even during aging. Results reflected by pH, specific gravity, %Bx, %ABV and DPPH assay (free radical scavenging property) on aging of CJPW (Fig. 8) were clear indicatives of metabolic changes which helped to determine the progress and completion of fermentation. Figure 8 demonstrated that the fermentation rate of CJPW was at its peak during the first 15 days. After 60 days of aging the alterations were apparently stopped indicating a slowing down fermentation progress or end to the metabolic process and reactions inside the broth. In the language of brewing, this stabilization point is often termed as lagering phase. Majumder, Saha, et al. (2021) have remarked probable reasons behind the lagering phase i.e., depletion of carbon source inside the broth and stationary phase attained by the yeast cells.

Results of qualitative tests and TPC assay revealed high phenolics in CJPD compared to the fermented sample signifying complete breakdown of phenolics during fermentation which was further validated by GC-MS results. A large peak of phenolic compound quinic acid was detected in CJPD while CJPW was rich with its breakdown forms as described below. Fermented product CJPW indicated low fatty acid content and it was further validated by GC-MS analysis as compounds like carbonyl component methylglyoxal, valeraldehyde, acetone derivatives etc. were found in noticeable amount which was possibly formed from lipids upon oxidation (Shibamoto 2015). Phenylethyl alcohol or PEA is a natural glycoside and which was the reason behind denotation of high glycosides in CJPW followed by CW.

In DPPH assay, antioxidant molecules act as a proton donor where the free radical is scavenged as the absorbance of DPPH solution is decreased thereby rendering a change in colour (Manivasagan et al. 2015). CJPW exhibited significantly higher (*P* < 0.05) antioxidant activity (DPPH assay and iodometric assay) compared to other samples as it was further found to be rich in potential antioxidative wine volatiles such as 2-hydroxy-GBL, DDMP (Majumder, Saha, et al. 2021), 2,3-butanediol (Shi et al. 2018), PEA (Chou et al. 2009), tyrosol (Covas et al. 2003) etc. Antioxidant (DPPH) assay on column chromatographic fractions of CJPW revealed diethyl ether as promising solvent followed by acetone and methanol. Interestingly, antioxidant compound GBL; 2,3-butanediol; PEA; and tyrosol are known to be soluble in organic solvents like diethyl ether and acetone and DDMP and tyrosol are also soluble in methanol. Therefore, results of column chromatography indicated a proper separation of antioxidant components.

Lipid peroxidation inhibition by unfermented decoction was higher than the fermented wine. Presence of hepatoprotective compound quinic acid (Kim et al. 2007) as a major compound in CJPD is the possible reason behind this result. Kalaiselvan et al. (2016) reported attenuation of hepatic oxidative stress by hydroxytyrosol and tyrosol. Lip (2014) reported hepatoprotective activity of dihydroxyacetone or DHA (CJPW’s major compound).

*Camellia japonica* flower particularly was not reported for antidiabetic activity earlier however, this GC-MS analysis has revealed antidiabetic compounds in its wine. Major CJPW compound DHA (Cathcart & Markowitz 1927) and tyrosol (Chandramohan et al. 2015) reportedly exhibit antidiabetic activity. Results of antidiabetic assay suggests that, unlike ordinary fermentation metabolites of *Saccharomyces cerevisiae*, high phenolics of *Camellia japonica* and the degraded phenolics (after fermentation) can be useful against diabetes.

Quinic acid, a shikimic acid pathway derived phytochemical, is the only phenolic compound found in our sample CJPD. Being one of the major precursors of many known bioactive phytochemicals and synthetic drugs, quinic acid, itself is rich in several medicinal values, such as anti-viral (including anti- hepatitis B, anti-herpes, anti-parainfluenza type 3, SARS-Cov-2); antimicrobial; and anti-inflammatory properties (Lingwan et al. 2021; Özçelik et al. 2011; Wang et al. 2009). Except quinic acid, peaks of terpenoids, long chain hydrocarbons like fatty acids, alkanes or waxes were detected which are generally found in cuticle of any flower petal. A number of antioxidant compounds (squalene; vitamin E; 2,3-dihydro-3,5-dihydroxy-6-methyl-4H-pyran-4-one or DDMP; eucalyptol etc.) were found in CJPD. Interestingly, bioactive compounds like eucalyptol; alpha-gurjunene; beta-caryophyllene; squalene; vitamin E etc. are known to exhibit anti-inflammatory activity and reported as major natural anti-inflammatory terpenoids of *Camellia japonica* leaf (Majumder et al. 2020).

Regarding other biological activities, Uzcátegui et al. (2007) reported antiproliferative effect of DHA against *Trypanosoma brucei* which indicates an antiprotozoal potential of the compound. Another abundant CJPW metabolite, methylglyoxal also possesses antimicrobial activity (Atrott & Henle 2009) which has been reported as an anticancer compound also (Talukdar et al. 2008). Phenolic metabolite tyrosol is an antioxidant and anti-inflammatory compound exerts its beneficial effects against hypertension, atherosclerosis, coronary heart disease, chronic heart failure, insulin resistance and obesity (Karković Marković et al. 2019). Having a wide range of bioactivity i.e., gastroprotective (anti-ulcer), antioxidant, antimicrobial and anti-inflammatory (analgesic and anti-edematous) activities (Majumder, Saha, et al. 2021), GBL (2-hydroxy-GBL and 2-acetyl-2-hydroxy-GBL) is the most abundant bioactive metabolite of CJPW. Methyl L-pyroglutamate (pGlu) is another bioactive metabolite of CJPW as analogues of pGlu possesses antifungal, neuritogenic, antibacterial and anti-inflammatory activities (Gang et al. 2018). pGlu enhances brain function due to its role in supporting the healthy production of neurotransmitters. pGlu is often used to support memory and learning in addition to managing anxiety (https://www.xtend-life.com/blogs/supplement-ingredients/l-pyroglutamic-acid).

Sugar fermented product glycerin was found to be the most abundant compound in fermented samples, i.e., 16.88% in CJPW and 86.43% in CW. Glycerin is a typical fermented product that is usually present every fermented alcoholic beverage around the world. Some of the other compounds of CJPW such as DHA or glycerone (11.55%) and glyceraldehyde (2.88%) are metabolites of the same pathway where glycerin is formed i.e., yeasts glycerol biosynthesis pathway (Tulha et al. 2012). Shibamoto (2015) demonstrated DHA, glyceraldehyde, methylglyoxal, acetic acid etc. as results of sugar’s oxidative degradation (or fermentation). Some byproducts of glycerin have also been found in fermented samples such as cis-5-Hydroxy-2-methyl-1,3-dioxane (1.27% in CW and 0.27% in CJPW) and 4-hydroxymethyl-2-methyl-1,3-dioxolane (0.5% in CW). These two components are derivatives of 1,3-formalglycerin (https://pubchem.ncbi.nlm.nih.gov/compound/78475) and glycerin formal (https://pubchem.ncbi.nlm.nih.gov/compound/21618) respectively. PEA is another yeast metabolite detected 1.73% in control whereas 3.03% in the petal wine. A variety of floral essential oils (including rose, carnation, hyacinth, Aleppo pine, orange blossom, ylang-ylang, geranium, neroli, and champaca) contain this compound. This compound is also a major aroma component of Muscat wine and responsible for honey-like aromas (http://www.ymdb.ca/compounds/YMDB01072). Antimicrobial properties of PEA are also reported (Chou et al. 2009). 2,3-Butanediol is another similar anaerobic fermentation product (Ng et al. 2012) which was found in control wine as a major compound with 7.52% peak area. In CJPW this compound was also detected in less amount but as three different derivatives i.e., 2,3-butanediol (0.98%), 2,3-butanedione, monooxime (1.85%) and 1,2,3-butanetriol (1.6%). Gastroprotective (anti-ulcer) and antioxidative agent GBL (detected as 2-hydroxy-GBL and 2-acetyl-2-hydroxy-GBL in CJPW) is a typical and major component of red wine reported by Vose et al. (2001) and also detected in tea petal wine (Majumder, Saha, et al. 2021). pGlu was reported to be detected in a broad range of wine samples and probiotic fermented foods (Aiello et al. 2022; Pfeiffer & König 2009). pGlu is a bioactive glutamine derivative that plays a key role in preserving the quality and nutritional value of foods. It is a five-membered lactam that is formed from glutamic acid through enzymatic and nonenzymatic pathways. Production of pGlu might depend on the starter microflora rather than on the substrate because glutamine cyclase and pyrrolidone carboxyl peptidase are the responsible enzymes that are released by microbial cultures, such as *Lactobacillus helveticus*, *Lactobacillus delbrueckii* subsp. *bulgaricus*, *Lactobacillus lactis* and *Streptococcus thermophiles* (Aiello et al. 2022). Valeraldehyde or pentanal (3.66% in CJPW) is another fermentation metabolite commonly found in fermented foods and beverages, mainly in aged beer (Smit et al. 2009). Valeraldehyde or pentanal was either derived from sugar or fatty acid after fermentation of CJPD (Shibamoto 2015). Hayashi et al. (2014) have reported antibacterial potential of this compound. Rhamnitol is generally (2,5-methylene-D,L-rhamnitol and 2,5-monomethylene-L-rhamnitol) is a secondary fatty sugar alcohol or alditol probably biosynthesized due to fermentation of various sugars of petals. Antimicrobial compound 3-deoxy-d-mannoic lactone (2.33% in CJPW) is reported as a fungal metabolite (Jideani et al. 2021; Sharma et al. 2016). Methylglyoxal is a Milliard reaction product. Formation of carbonyl compound methylglyoxal from lipids or fatty acid was reported (Shibamoto 2015). Oxidation of DHA (detected with 11.55% peak area) can also be responsible for the synthesis of methylglyoxal (Lip et al. 2013). DDMP is another fungal secondary metabolite and potential antioxidative and antifungal agent (Majumder, Saha, et al. 2021). Majumder, Saha, et al. (2021) reported this compound in tea petal wine as a possible derivative of a flavonoid biosynthesized during fermentation by yeast. However, adequate peak area of this compound in the unfermented decoction (CJPD) also indicates occurrence of Milliard reaction (a non-enzymatic reaction) that often happens with high temperature treated foods (Chen et al. 2021). Last but not least, breakdown of CJPD’s major compound quinic acid had to be a major concern in this study as no trace of quinic acid was found in CJPW indicating a complete degradation or utilization of the compound during fermentation. Metabolomics based probable quinic acid degradation has been described below.

**Fig. 9:**
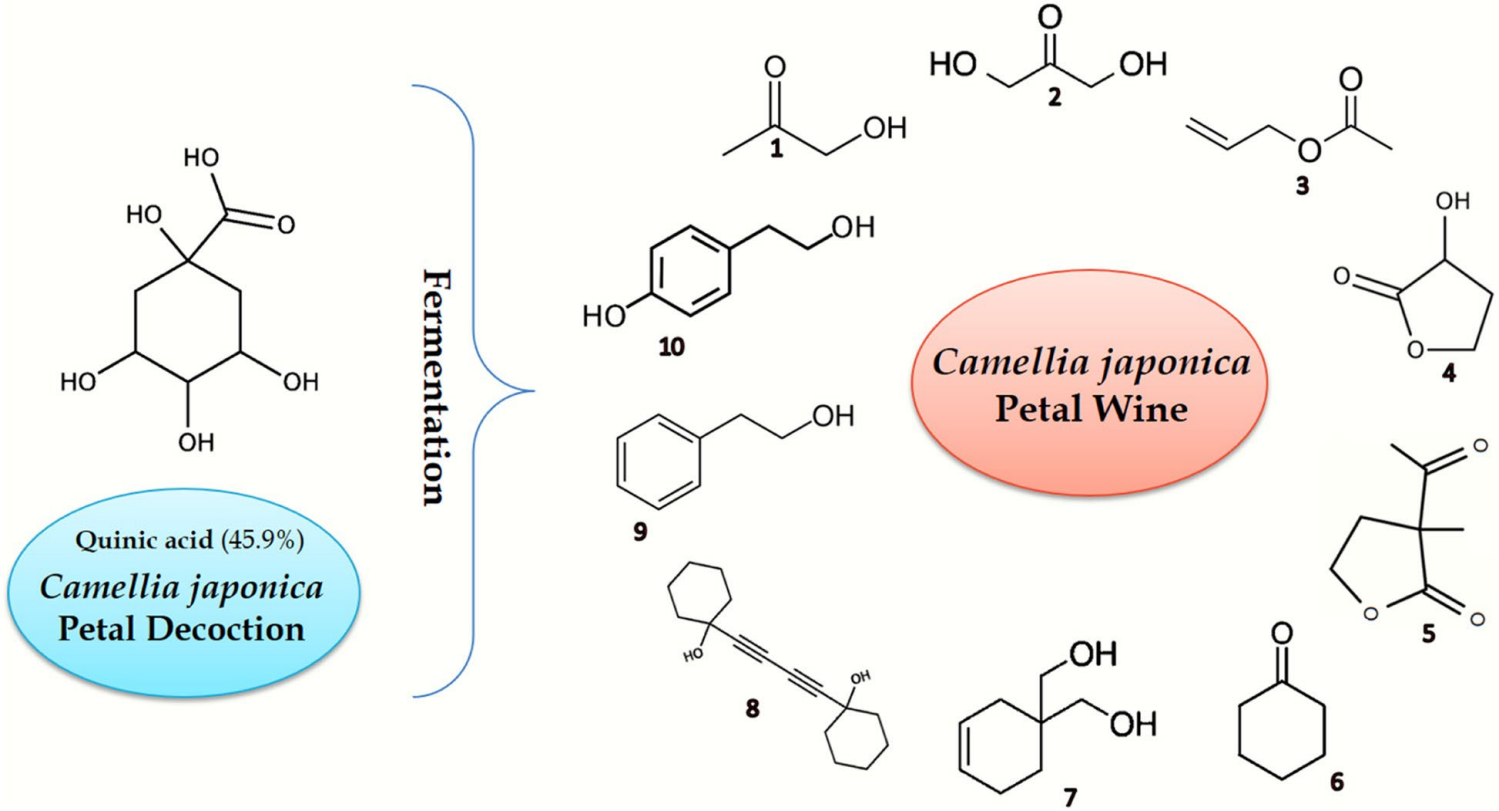
Proposed biosynthesis pathway of certain CJPW metabolites formed after fermentation-led oxidation of CJPD’s major compound quinic acid. 1: 1-hydroxyacetone (2.96%); 2: dihydroxyacetone (11.55%); 3: acetic acid, 2-propenyl ester (2.46%); 4: 2-hydroxy-gamma-butyrolactone (13.33%); 5: 2-acetyl-2-hydroxy-gamma-butyrolactone (0.27%) 6: cyclohexanone (0.58%); 7: 3-cyclohexene-1,1-dimethanol (1.02%); 8: 1,1′-butadiynylenedicyclohexanol (2.56%); 9: phenylethyl Alcohol (3.03%); 10: tyrosol (1.11%)

Hulme and Arthington (1953) did pioneer research on quinic acid breakdown. According to their report, quinic acid, after successive oxidation processes, produces intermediate compound acetonedicarboxylic which is further converted into malonic acid. Malonic acid on oxidation breaks down to acetic acid (Hulme & Arthington 1953). Detection of acetic acid (esterified) in CJPW with a considerable peak area (2.46%) suggests oxidative degradation of CJPD’s quinic acid during fermentation. Shibamoto (2015) reported degradation of quinic acid into several volatile compounds which have been detected as CJPW’s metabolite validating the fermentation or oxidation of substrate’s major compound (Fig. 9). Volatiles such as furans, cyclohexanones and aromatic compounds are formed as moieties of quinic acid via dehydration (Shibamoto 2015). Furans like derivatives of GBL (a furanone); cyclohexanones such as cyclohexanone (0.58%), 3-cyclohexene-1,1-dimethanol (1.02%) and cyclohexanone dimer i.e., 1,1′-butadiynylenedicyclohexanol (2.56%); and phenolic antioxidant tyrosol (1.11%) are those CJPW’s metabolites which were some of the possible breakdown products of CJPD’s quinic acid. Moreover, CJPW’s metabolite 1-hydroxyacetone or 2-propanone, 1-hydroxy- (2.96%) was formed due to oxidation of quinic acid (Shibamoto 2015). Perhaps, the dimer of this compound i.e., major compound dihydroxyacetone (11.55%) was also formed following the same pathway after successive steps of oxidation. Phenolic glycoside PEA (also the precursor and phenolic analogue of tyrosol) might also be synthesized from quinic acid just like tyrosol. Detection of this compound in control wine (CW) with a notable peak area (1.73%) might deny this possibility, but the compound’s upsurge in the fermented product (3.03%) supports the quinic acid breakdown concept. Chemical structures of these ten compounds, biosynthesized from quinic acid during fermentation, have been graphically represented in Fig. 9 for a better understanding.

Study on flavour chemistry of the CJPW revealed that numerous metabolites of CJPW are cumulatively responsible for the distinct wine like flavour of CJPW. DHA is responsible for minty odour and sweet flavour while both glycerin and glyceraldehyde work as natural sweeteners in foods and beverages. PEA generates a pleasant floral odour which occurs widely in nature. GBL is responsible for the creamy and fruity odour and peach-like taste of wine. Aged beer compound valeraldehyde is responsible for wine like flavour. 3-Pentanol, 2,3,4-trimethyl- exhibits fruity odour. Major compound methylglyoxal (13.25%) is one of such compounds that produces caramellic flavour in wine. pGlu has been detected as a methyl ester in CJPW. This esterification actually happens during fermentation that releases a fermented aroma (Pfeiffer & König 2009). Jideani et al. 2021 reported that 3-deoxy-d-mannoic lactone contributes to the flavour of African traditional fermented beverage “Kunu-zaki” during fermentation. DHA; glycerin; glyceraldehyde; PEA; GBL; 3-Pentanol, 2,3,4-trimethyl-; methylglyoxal; pGlu; and 3-deoxy-d-mannoic lactone have summative contribution towards the wine like flavor of CJPW.

## Conclusion

This petal wine was an outcome of metabolic pathways of brewer’s yeast and several other reactions, which converted the metabolites of *Camellia japonica* flower petals and added sugar during fermentation. The key outcomes of this research were detection of quinic acid in the substrate i.e., *Camellia japonica* petal decoction and fermentation-led breakdown of the compound in the wine. The breakdown was also validated by metabolomics through a proposed quinic acid degradation pathway. However, further research is needed to discover the enzymes responsible for the degradation. Being a source of quinic acid and other bioactive compounds like DDMP; eucalyptol; alpha-gurjunene; beta-caryophyllene; squalene; alpha-spinasterol; vitamin E etc., this flower can be used by pharmaceuticals or in development of formulations like herbal tea and herbal medicines etc. beside producing fermented beverages.

## Data Availability

All data analysed during this study are included in this article.

## Abbreviations

GC-MS: Gas chromatography-mass spectrometry
CJPD: *Camellia japonica* petal decoction
CJPW: *Camellia japonica* petal wine
CW: Control wine
DPPH: 2,2-Diphenyl-1-picrylhydrazyl
OD: Optical density
%T: Percentage transmission
ABV: Alcohol by volume (%)
GBL: Gamma-Butyrolactone
DDMP: 2,3-Dihydro-3,5-dihydroxy-6-methyl-4H-pyran-4-one
DHA: Dihydroxyacetone
PEA: Phenylethyl alcohol
PGlu: L-Pyroglutamic acid
